# Assessing the Validity and Reproducibility of an Iron Dietary Intake Questionnaire Conducted in a Group of Young Polish Women

**DOI:** 10.3390/nu9030199

**Published:** 2017-02-27

**Authors:** Dominika Głąbska, Dominika Guzek, Joanna Ślązak, Dariusz Włodarek

**Affiliations:** 1 Department of Dietetics, Faculty of Human Nutrition and Consumer Sciences, Warsaw University of Life Sciences (WULS-SGGW), 159c Nowoursynowska Str., 02-776 Warsaw, Poland; joanna_slazak@sggw.pl (J.Ś); dariusz_wlodarek@sggw.pl (D.W.); 2 Department of Organization and Consumption Economics, Faculty of Human Nutrition and Consumer Sciences, Warsaw University of Life Sciences (WULS-SGGW), 159c Nowoursynowska Str., 02-776 Warsaw, Poland; dominika_guzek@sggw.pl

**Keywords:** iron, food frequency questionnaire, validation study, validity, reproducibility, young women

## Abstract

The aim of the study was to analyse a designed brief iron dietary intake questionnaire based on a food frequency assessment (IRONIC-FFQ—IRON Intake Calculation-Food Frequency Questionnaire), including the assessment of validity and reproducibility in a group of 75 Polish women aged 20–30 years. Participants conducted 3-day dietary records and filled in the IRONIC-FFQ twice (FFQ1—directly after the dietary record and FFQ2—6 weeks later). The analysis included an assessment of validity (comparison with the results of the 3-day dietary record) and of reproducibility (comparison of the results obtained twice—FFQ1 and FFQ2). In the analysis of validity, the share of individuals correctly classified into tertiles was over 50% (weighted *κ* of 0.36), while analysis of correlation revealed correlation coefficients of almost 0.5. In the assessment of reproducibility, almost 80% of individuals were correctly classified and less than 3% were misclassified (weighted *κ* of 0.73), while a correlation coefficient higher than 0.85 was obtained. Both in the assessment of validity and of reproducibility, a Bland–Altman index of 6.7% was recorded (93.3% of compared pairs of results were in the acceptable range, attributed to differences within ± 2SD limit). Validation of the IRONIC-FFQ revealed a satisfactory level of validity and positively validated reproducibility.

## 1. Introduction

Anemia has been indicated by the World Health Organization (WHO) as one of the main diet-related health risks, while iron deficiency was stated to be among the most important contributors to the global burden of the disease [1]. It is indicated that about 50% of anemia cases is attributed to iron deficiency [2]. This is a global problem, as a general prevalence of 23%–27% is recorded for anemia, which corresponds to 1620 million affected individuals [3]. At the same time, it is supposed that if anemia prevalence exceeded 20%, some degree of iron deficiency would be present in about 50% of the population [4].

A 50% reduction of anemia in women of reproductive age is formulated as the WHO’s global target for the year 2025 [5], since the highest frequency of anemia is recorded in women of reproductive age and in pre-school children [6]. Moreover, a reduction of anemia prevalence in women of reproductive age would improve not only their health but also the health of their offspring, as the results of a meta-analysis have indicated a strong association between maternal iron-deficiency anemia and adverse birth outcomes [7].

Currently, inadequate iron intake is observed in the case of 16%–19% of young Polish women and it is a group of the highest frequency of inadequate intake [8], as in some studies inadequate iron intake is observed even for 97% of them [9]. At the same time, the share of non-pregnant young women, with blood haemoglobin concentration lower than 120 g/L is, in Poland, on a level of 23% [2]. In comparison with other countries, it must be emphasized that the incidence of iron deficiency and iron deficiency anemia is, in developing countries, higher than in the industrialized ones [10] and the highest share of non-pregnant young women with blood haemoglobin concentration lower than 120 g/L is 56% for Senegal [2]. Taking into account the lack of success in reaching the target of reduced anemia prevalence that is indicated so far, some authors have suggested that it may be impossible to reach the target by the year 2025 [11].

To reduce anemia prevalence, it is necessary to conduct screening in at-risk groups and to find deficient individuals, as was recommended by the National Screening Committee of the United Kingdom [12]. Simultaneously, in a programme conducted by the Micronutrient Initiative, the United Nations International Children’s Emergency Fund, the Canadian International Development Agency, the United Nations Food and Agriculture Organization (FAO), the United Nations Standing Committee on Nutrition, and the WHO, it was indicated that in the case of iron-deficient individuals, dietary iron assessment, counselling, monitoring and support should be conducted and provided in order to obtain improvement of iron intake [13]. Such a nutritional approach has been indicated as one of the effective ways to combat iron-deficiency anemia [14]. As the food frequency questionnaire has been stated to be one of the possible methods to assess nutrients intake [15], it may be supposed that it is a useful tactic to provide dietary iron advice also in the case of iron-deficient individuals.

The aim of the presented study was to assess the validity and reproducibility of the designed iron dietary intake questionnaire based on a food frequency assessment (IRONIC-FFQ—IRON Intake Calculation-Food Frequency Questionnaire) in a group of Polish women aged 20–30 years. The aim of the validation was to obtain the iron-specific tool that would enable quick assessment of iron intake in the group of young women.

## 2. Materials and Methods

The study was conducted according to the guidelines laid down in the Declaration of Helsinki. The Ethics Committee of the Regional Medical Chamber in Warsaw, Poland, approved the study protocol (No. 4/08; 7.02.2008) and all participants provided written informed consent.

### 2.1. Designing an Iron Dietary Intake Questionnaire (IRONIC-FFQ—IRON Intake Calculation-Food Frequency Questionnaire)

The designed IRONIC-FFQ was based on a food frequency assessment. Only food products that were sources of iron were taken into account. Due to the fact that the questionnaire was designed for a group of young women (as characterised by the highest frequency of anemia), the fortified food products dedicated for other groups (e.g., for children) were not included. During the design of the IRONIC-FFQ, information about main sources of iron in the Polish diet was taken into account—it is indicated that the main sources of iron in the analysed group are cereal products, meat/fish and meat/fish products, vegetables, potatoes and fruits [16]. Moreover, all other food products that are also characterised by iron content no lower than 0.1 mg per 100 g were chosen on the basis of Polish food composition tables [17].

All food products meeting the assumed criteria were grouped into 12 food product groups and 32 related sub-groups characterised by a similar range of iron content, as presented in Table 1. The clustering procedure (combining products of the same food product sub-group characterised by a similar iron content and presenting them as a sub-group instead of a list of single products) was conducted to obtain a lower number of items included in the questionnaire. In the group of fortified food products available on the Polish market, mainly corn flakes and cereal products are iron-fortified [18], thus only such products were included in the IRONIC-FFQ.

The most popular serving sizes were determined on the basis of the Polish food model booklet [19] and verified during the pilot research. The pilot study was conducted on a group of five young female individuals who received the IRONIC-FFQ, including preliminarily specified portion sizes of food products and dishes. The participants were asked to fill in the questionnaire. Subsequently, portion sizes of the products and dishes were verified and, if needed, changed into more reasonable sizes on the basis of the obtained declared numbers of servings.

For each group of food products, the average iron content in a serving was specified, as is presented in Table 1 [17]. Information about the iron quantity in the serving was not placed in the IRONIC-FFQ in order to not interfere in providing the answers, but the serving sizes were specified in the IRONIC-FFQ.

Individuals were asked about the exact number of servings of products from the groups specified in the IRONIC-FFQ which had been consumed during a typical week throughout the previous year (open-ended question). They were asked to indicate the servings of products consumed and added to consumed dishes. In the questionnaire, the participants declared the typical number of servings of each product (being able to indicate not only whole integers but also decimal parts).

During the analysis, the number of servings was divided into 7 days a week to obtain the daily number of servings. The iron intake from the products was estimated using the following formula: iron intake (mg) = daily number of servings × typical iron content in one serving (Table 1). The total daily iron intake was obtained as the sum of the values of iron intake from all groups of products.

For the meat and meat products, an additional question was put in the IRONIC-FFQ—the participants were asked to indicate up to the five most frequently chosen types of meat and up to the five most frequently chosen types of meat products. If such a product/products for a group was/were specified, instead of the typical iron content in one serving, as presented in Table 1, an individualized iron content in the serving was taken into account during the calculation for each participant. The individualized calculated iron content in the serving was obtained as a mean for the most commonly chosen products from a group. As a consequence, the iron content was assessed more precisely for the meat and meat products than for the other products, as meat and meat products were observed as possibly being the main source of error in the iron intake estimation.

### 2.2. Validation of the IRONIC-FFQ

The IRONIC-FFQ was validated in a group of young women. The invitation to participate in the validation of the IRONIC-FFQ as well as information about the inclusion criteria were distributed via social media. The inclusion criteria were as follows: women, aged 20–30 years, not undergoing body mass reduction or on any special diet, not pregnant and not during lactation, without any chronic diseases, and living in Warsaw. There were 87 individuals meeting the inclusion criteria who volunteered to participate in the study. Finally, validation of the assessed IRONIC-FFQ was conducted in a group of 75 young women, as 12 of the individuals who had initially volunteered did not complete all of the required elements. It must be indicated that the main limitation of the conducted validation study was the fact that the sample was not random, so individuals who had volunteered for the study were probably motivated to properly conduct the 3-day dietary record and to fill in the IRONIC-FFQ, but such a situation is a limitation in many similar studies [20].

The sample size for the validation study was calculated on the basis of data regarding the number of women aged 20–30 years in Poland (3,109,814 individuals in December 2013 on the basis of data from the 2014 Demographic Yearbook of Poland [21], the prevalence of iron intake inadequacy (10% in most of the nutritional surveys on the basis of the *EURopean micronutrient RECommendations Aligned (EURRECA) Network of Excellence Project* [22]), the assumed confidence level of 95% and the assumed maximum error of the iron intake adequacy assessment of 10%. For the preconceived conditions, the minimum sample size was calculated as 35 individuals. The sampling procedure for validation was conducted as for the previous paper, while the number of participants was at an equal level [23].

The study of validation was conducted in autumn, during a period of 3 months—from September to November 2014. Despite the fact that chemically assessed iron contents in Polish diets are in general similar during the four seasons of the year [24], it was decided to conduct the study during one season. The recommendations on the assessment of food frequency questionnaires’ reproducibility specify that an interval between repeat measurements should be chosen to minimise changes over time [25]. During this period, the participants were asked to conduct 3-day dietary records in printed form and to fill in the IRONIC-FFQ twice (FFQ1—filled in directly after conducting the 3-day dietary record and FFQ2—filled in 6 weeks after the FFQ1), also in printed form.

Validation of the obtained IRONIC-FFQ questionnaire was conducted according to methodology published previously [23]. It included an analysis of the validity (external validation comparing results of the FFQ1 with results of the 3-day dietary record, whereas both assessments were conducted by the same researcher) and reproducibility of the method (internal validation comparing results obtained twice—FFQ1 and FFQ2, with both assessments conducted by the same researcher), as defined by Willett and Lenart [26]. Both the 3-day dietary record and the IRONIC-FFQ assessments were based on self-reported data.

For the 3-day dietary record, the basis of the analysis was the record conducted in three typical random and not successive days (2 weekdays and 1 day of the weekend). The dietary record was conducted on the basis of widely accepted and applied rules—using a structured format, with additional questions about name of the meal, time and location of consumption, meal ingredients and weight of serving (while weighted using kitchen scale) or size of serving (while estimated using standard household measures) [27]. To provide reliable estimates of food intake, participants were instructed on the principles of making the dietary record as well as on the necessity of accurate and scrupulous recording of all food products consumed and beverages drunk, while the serving sizes were verified afterwards by a dietitian using the Polish food model booklet [19]. The iron intake was analysed using Polish dietician software—“Dietetyk 2” (National Food and Nutrition Institute, 2001) and the Polish database of the nutritional value of products [17].

### 2.3. Statistical Analysis

The statistical analysis of validation included five elements:

(1) Calculation of root mean square errors of prediction (RMSEP) and the median absolute percentage errors (MdAPE) of iron intake in the assessment of validity (FFQ1 vs. 3-day record) and of reproducibility (FFQ1 vs. FFQ2).
(2) Assessment of the share of individuals classified into the same tertile and misclassified (classified into opposite tertiles) in the assessment of validity (FFQ1 vs. 3-day record) and of reproducibility (FFQ1 vs. FFQ2).
(3) Calculation of the weighted κ statistic with linear weighting to indicate the level of agreement between the classifications into tertiles in the assessment of validity (FFQ1 vs. 3-day record) and of reproducibility (FFQ1 vs. FFQ2)—according to the criteria of Landis and Koch [28], values <0.20 were treated as slight agreement, 0.21–0.40—as fair, 0.41–0.60—as moderate, 0.61–0.80—as substantial, and 0.81–1.0—as almost perfect agreement.
(4) Analysis of the correlations between results obtained in the assessment of validity (FFQ1 vs. 3-day record) and of reproducibility (FFQ1 vs. FFQ2)—the normality of distribution of the results was analysed using the Shapiro–Wilk test and then Spearman’s rank correlation was applied for nonparametric distribution.
(5) Analysis of the Bland–Altman plots in the assessment of validity (FFQ1 vs. 3-day record) and of reproducibility (FFQ1 vs. FFQ2)—the results were interpreted using the Bland–Altman index, whereas the limits of agreement value (LOA) was calculated as the sum of the mean absolute differences of iron intake measured by the two methods, and the ± standard deviation of the absolute difference of iron intake recorded for the two methods magnified by 1.96. In the analysis conducted with the Bland–Altman method to assess agreement between the measurements, a Bland–Altman index of a maximum of 5% (95% of individuals observed to be beyond the LOA) was interpreted, as commonly assumed [29], as positive validation of the method of measurement.

Moreover, for each participant, the calculated iron intake obtained using the IRONIC-FFQ, conducted twice (FFQ1, FFQ2), and the 3-day dietary record were compared with the recommended intake (8 mg) according to Polish recommendations on the Estimated Average Requirement (EAR) level [30]. For each method, the individuals were classified into categories of either adequate or inadequate intake. In a comparison of the two methods (FFQ1 vs. 3-day record; FFQ1 vs. FFQ2), the shares of individuals of the same category and of the conflicting iron intake adequacy category were assessed. The same category was interpreted as adequate intake for both methods or as inadequate intake for both methods, while the conflicting category was interpreted as the opposite intake (adequate intake and inadequate intake) for the two assessed methods.

The level of significance was accepted as *p* ≤ 0.05. Statistical analysis was carried out using Statistica software version 8.0 (StatSoft Inc., Tulsa, OK, USA) and Bland–Altman Statistica software macro by Matt Coates, version 2009 (StatSoft Inc., Tulsa, OK, USA).

## 3. Results

Iron intake observed in the analysed group, calculated using the 3-day dietary record and IRONIC-FFQ conducted twice (FFQ1, FFQ2), is presented in Table 2. In the analysed group of young women, about 5–10 min was needed to complete the paper IRONIC-FFQ form, with 32 questions about product groups and two additional questions about most frequently chosen types of meat and meat products, included. The observed iron intake for the majority of the analysed group was stated to be adequate, since for each applied assessment over 60% of the group was characterised by intake that was higher than the Estimated Average Requirement level of 8.0 mg a day.

The calculated contribution of product groups into the daily iron intake obtained from the IRONIC-FFQ conducted twice is presented in Table 3. It was observed that the main product group contributing to iron intake was the group of cereal products, responsible for over 30% of iron dietary intake.

**Figure 1:**
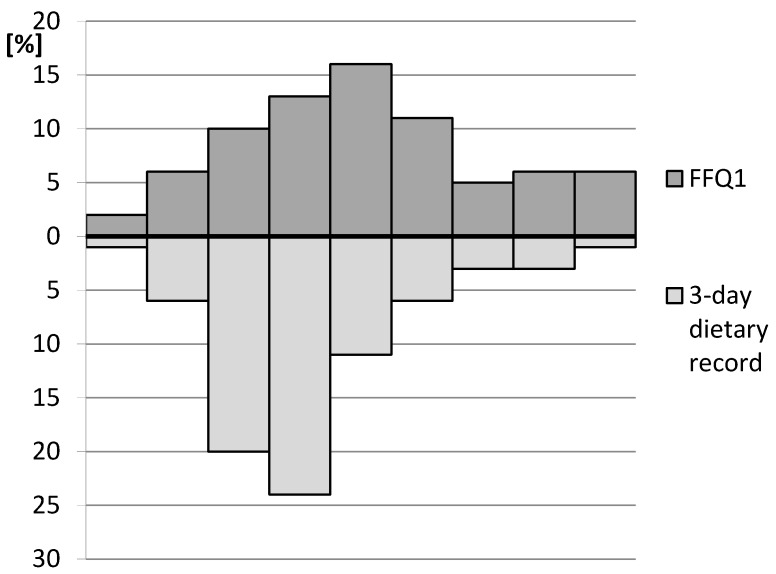
The histogram displaying the distribution of iron intake estimated on the basis of the 3-day dietary record and the analysed IRONIC-FFQ1.

The calculated RMSEP of iron estimation in the assessment of validity in comparison with the results of the 3-day dietary record for the analysed IRONIC-FFQ was 5.3 mg. Simultaneously, the MdAPE of iron intake for a comparison with the 3-day dietary record was 27.6%. The histogram displaying the distribution of iron intake estimated on the basis of the 3-day dietary record and the analysed IRONIC-FFQ1 is presented in Figure 1.

**Figure 2:**
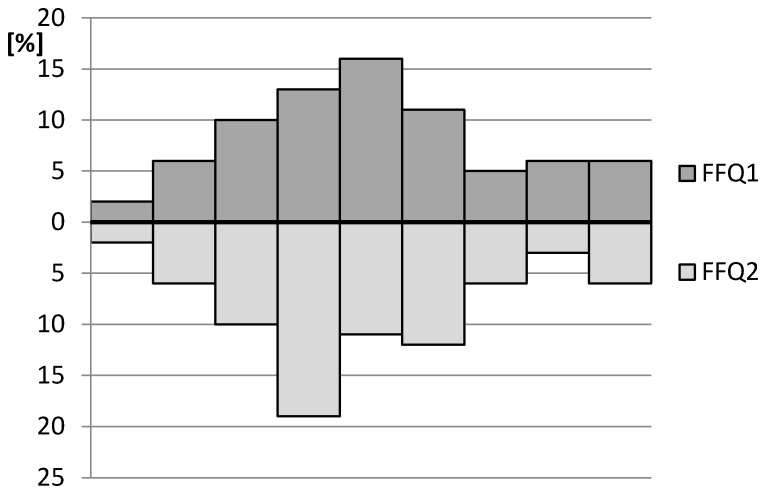
The histogram displaying the distribution of iron intake estimated on the basis of the IRONIC-FFQ1 and IRONIC-FFQ2.

In the assessment of reproducibility, the RMSEP of iron estimation—while the food frequency assessment was conducted twice during a period of 6 weeks—was 2.3 mg. The MdAPE of iron intake for the comparison between the FFQ1 and FFQ2 was 7.7%. The histogram displaying the distribution of iron intake estimated on the basis of the IRONIC-FFQ1 and IRONIC-FFQ2 is presented in Figure 2.

The share of individuals classified into the same tertile in the validation of the IRONIC-FFQ as well as the weighted κ statistic are presented in Table 4. The higher share of individuals classified into the same category (78.7%), accompanied by the lower share of misclassified individuals (2.7%), was stated for the FFQ1 vs. FFQ2 comparison (assessment of reproducibility). Simultaneously, in the assessment of reproducibility, the weighted *κ* statistic indicated substantial agreement, while in the assessment of validity it indicated fair agreement.

The correlation was analysed between the FFQ1 and the 3-day dietary record daily iron intake. The Spearman rank correlation coefficient revealed a statistically significant association (*R* = 0.48) between daily iron intake obtained using the verified method of the IRONIC-FFQ (FFQ1) and the 3-day dietary record, while 23.04% of the variation was explained by the regression line.

The correlation between the FFQ1 and FFQ2 daily iron intake was also analysed. In the assessment of reproducibility, similarly as for the assessment of validity, the Spearman rank correlation coefficient revealed a statistically significant association (*R* = 0.87) between daily iron intake obtained using the IRONIC-FFQ for FFQ1 and FFQ2, while 75.69% of the variation was explained by the regression line.

**Figure 3:**
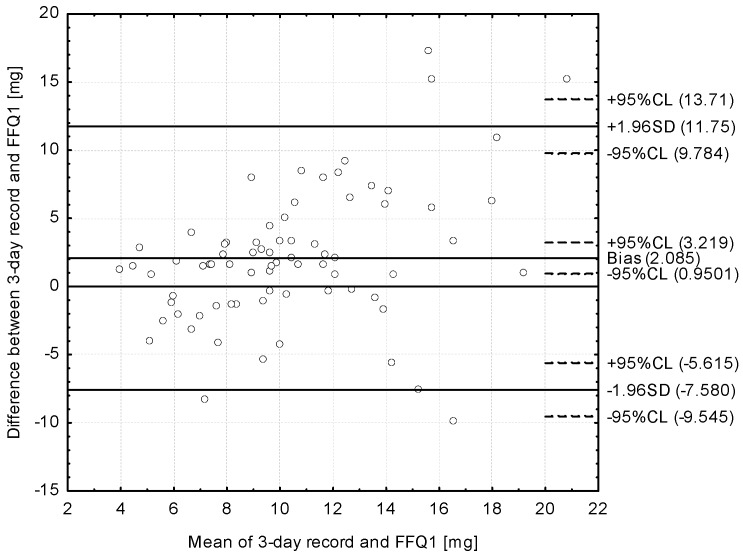
Bland–Altman plot comparing the IRONIC-FFQ1 with the 3-day dietary record iron daily intake (Bland–Altman index of 6.7%); FFQ1—IRONIC-FFQ food frequency questionnaire filled out directly after conducting the three-day dietary record.

The Bland–Altman plot comparing FFQ1 with the 3-day dietary record daily iron intake is presented in Figure 3. The mean absolute difference in iron intake was observed to amount to 2.085. After adding ±1.96 standard deviation for the LOA, an interval from −7.580 (lower agreement limit) to 11.750 (upper agreement limit) was obtained. The number of individuals observed to be beyond the LOA value was 70 out of 75, which confirmed a Bland–Altman index of 6.7% (93.3% of compared pairs of results were in the acceptable range, attributed to differences within the ±2SD limit).

**Figure 4:**
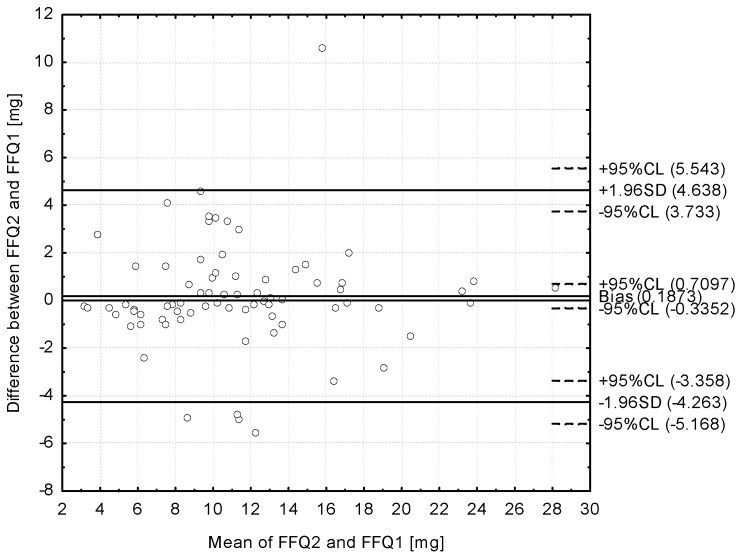
Bland–Altman plot comparing the IRONIC-FFQ2 with the IRONIC-FFQ1 iron daily intake (Bland–Altman index of 6.7%); FFQ1—IRONIC-FFQ food frequency questionnaire filled out directly after conducting the three-day dietary record; FFQ2—IRONIC-FFQ food frequency questionnaire filled out 6 weeks after the FFQ1.

The Bland–Altman plot comparing the FFQ1 with FFQ2 daily iron intake is presented in Figure 4. The mean absolute difference in iron intake was observed to amount to 0.1873. After adding ±1.96 standard deviation for the LOA, an interval from −4.263 (lower agreement limit) to 4.638 (upper agreement limit) was obtained. The number of individuals observed to be beyond the LOA value was 70 out of 75, which confirmed a Bland–Altman index of 6.7% (93.3% of compared pairs of results were in the acceptable range, attributed to differences within the ±2SD limit).

## 4. Discussion

Taking into account the WHO’s global target for the year 2025 of a 50% reduction of anemia in women of reproductive age [5], iron intake monitoring in large groups of young women may be indicated as a very important goal to achieve. As the possibilities to influence the iron absorption, or iron loss (especially influential in the analysed group, due to menstrual bleeding), are limited, influencing dietary iron intake is the key action to solve mentioned problem.

Despite the fact that a number of validation studies for food frequency questionnaires, including iron intake assessment, had been conducted and published, only a small number had been designed for iron exclusively. An analysis conducted for Australia and New Zealand [31] indicated that until April 2010, nine published food frequency questionnaires included iron, but only one was a brief iron-specific food frequency questionnaire [32]. However, the above-mentioned questionnaire of Heath et al. [32] was iron-specific but not designed exclusively for iron, as also other nutrients and products were included as its absorption modifiers. As a consequence, a total of 206 questions regarding food products intake sorted into 17 food product groups were in the questionnaire [32].

Some multiple-nutrients comprehensive food frequency questionnaires consist of many questions and are designed to assess the intake of a number of nutrients, such as the food frequency questionnaire applied by Barrett and Gibson [33], including 297 questions regarding food products to assess the intake of 37 nutrients. Such questionnaires may be of great value in the general ranking of an individual’s dietary intake in clinical practice, but the above-mentioned questionnaire, despite the good validation demonstrated for iron, overestimated the dietary intake in comparison with the food record [33]. Moreover, it has been indicated that while more questions about food products are included in the food frequency questionnaire, the risk of overestimation is higher [31].

For questionnaires in general, it is indicated that the response rate depends on the length of the form [34]; even if long questionnaires are not rejected by respondents but filled in, the answers may be uniform or inaccurate due to fatigue among the respondents, which may cause lower accuracy of the results depending on the time needed to fill in the questionnaire [35]. In the study of Fayet et al. [36], 235 questions were included, and the authors declared that about 45 minutes was needed to complete their food frequency questionnaire.

Taking into account the above-mentioned concerns associated with comprehensive food frequency questionnaires, related to a lot of questions, causing high risk of overestimation and low response rate, shorter iron-specific questionnaires may be needed in specific risk groups in order to monitor iron intake. As the group of women of reproductive age is indicated by the WHO as the specific group needing dietary support in order to obtain satisfying iron needs, to reduce anemia risk [5], the tool enabling gathering individuals characterised by insufficient intake would be of a great value.

An especially important issue in designing the IRONIC-FFQ was the number of included food products and questions. Due to the products clustering procedure, a list of 32 questions about sub-groups of food products was obtained and the time needed to complete the IRONIC-FFQ was reduced in comparison with suppositious time needed to complete a food frequency questionnaire including single products. Due to the fact that the aim of using the IRONIC-FFQ as presented here may be an assessment of iron intake in order to indicate individuals characterised by inadequate intake, a higher level of overestimation would contribute to concealing some individuals with inadequate intake and, as a consequence, would lead to an underestimation of their number. Moreover, the iron absorption modifiers, such as in the questionnaire of Heath et al. [32] were not included, to obtain the questionnaire as simple and specific as possible.

The number of questions included in the IRONIC-FFQ was low, i.e., 32 food item questions obtained due to clustering is less than for other iron food frequency questionnaires, as the main goal was to achieve quite a simple tool, even if its validity or repeatability were slightly lower. In the compared questionnaires of Beck et al. [10], Galante and Colli [37], and Sichieri and Everhart [38], there were 144, 79 and 73 questions included, respectively.

For the previously mentioned questionnaires, it was recognised that they had acceptable or good validation demonstrated for iron. The IRONIC-FFQ is shorter than the previously mentioned questionnaires, and it should be stated that shorter questionnaires, associated with quicker data acquisition, may also be characterised by lower accuracy of the results [39].

The above-mentioned validated food frequency questionnaires designed for iron were developed and validated in such countries as Australia [36], New Zealand [10,32], Canada [40], and Brazil [37], while for European populations the lack of a brief iron-only food frequency questionnaire can be observed. Taking into account the specific food products consumed in each geographical region, questionnaires dedicated to a specific country or region, as well as an ethnic group [41], should be designed which include these given products and should be validated in a subsample of the population in which they will eventually be used [32]. This has been indicated as an important aim for countries in which no dedicated food frequency questionnaires exist [42]. Especially when, as in the case of European women of reproductive age, anemia prevalence of 20.1% has been recorded and corresponds to 35.7 million affected individuals [2], the IRONIC-FFQ may be a useful tool in the dietary advice strategy of iron-deficient individuals. Taking this into account, the designed IRONIC-FFQ was elaborated including typical European food products and thus to enable application of the questionnaire in various European countries and regions.

The other authors applied various methods to validate their food frequency questionnaires, but the most commonly used were an analysis of the share of individuals correctly classified into tertiles/quartiles/quintiles and analysis of correlation.

Masson et al. [43] indicated that the validity of the food frequency questionnaire may be confirmed by more than 50% of subjects correctly classified and less than 10% of subjects grossly misclassified into tertiles. In the analysis of validity of the IRONIC-FFQ, the assessment of the share of individuals correctly classified into tertiles revealed over 50% for the comparison between the 3-day dietary record and FFQ1. At the same time, the assessment of the share of individuals misclassified was revealed as over 10%. In the assessment of reproducibility, almost 80% of individuals were correctly classified and less than 3% were misclassified for the IRONIC-FFQ. This was confirmed by the weighted κ statistic, indicating substantial agreement in the assessment of reproducibility. However, the level recommended by Masson et al. [43] for iron is not always obtained in the other validated questionnaires. It was obtained neither by Fayet et al. [36] nor by Heath et al. [32] for classification into quartiles. It was, at the same time, obtained by Barrett and Gibson [33] as well as by Beck et al. [10].

Masson et al. [43] also stated that the validity of the food frequency questionnaire may be confirmed by correlation coefficients above 0.5. At the same time, it has been indicated that coefficients higher than 0.7 are rare, so such a level is the so-called “ceiling of validity”, which is associated with the fact that the inherent complexity of the diet cannot be fully captured by a structured questionnaire [44]. In the assessment of validity of the IRONIC-FFQ, the analysis of correlation revealed a correlation coefficient of almost 0.5, while in the analysis of reproducibility of the IRONIC-FFQ, a correlation coefficient higher than 0.85 was obtained. It may be stated that the required value was obtained in the assessment of reproducibility in the present study. The fact that the required level was almost obtained in the assessment of validity but cannot be definitely stated should not disqualify the IRONIC-FFQ, as a level above 0.5 was also not obtained in the studies of Heath et al. [32], Galante and Colli [37], and Sichieri and Everhart [38].

Despite the prominent results of the tertile comparison and the analysis of correlation as presented above, the Bland–Altman plot, being the “gold standard” in the validation [45], was also analysed, as it is recommended that it should be used to assess reproducibility and validity, rather that correlation [46]. A Bland–Altman index of 5% may be interpreted as a positive validation of the method of measurement, as it is commonly assumed [29]; however, other authors have indicated that in the Bland–Altman plot, the LOA should not just clinically impact the obtained results [33]. Despite the fact that the level of 5% was not obtained in the presented validation of the IRONIC-FFQ, 6.7% may be indicated as such a level that may not influence the obtained results. Also, in the study of Beck et al. [10], the number of individuals observed to be beyond the LOA value was 111 out of 115 (Bland–Altman index of 3.5%) and 110 of 115 (4.4%) in the analysis of validity, as well as 110 out of 115 (4.4%) and 105 out of 115 (8.7%) in the analysis of reproducibility, for the “healthy” dietary pattern and the “sandwich and drinks” dietary pattern, respectively.

When analysing both the IRONIC-FFQ and the other presented validation studies of iron food frequency questionnaires, it may be stated that a questionnaire with a simultaneously high level of reliability and a low level of complexity may be difficult to obtain. None of the brief iron food frequency questionnaires designed so far can be indicated as a fully validated method of iron intake assessment taking into account the criteria of Masson et al. [43]. However, for the time being, some ready-to-use questionnaires designed for various populations exist, so they may be applied for the assessment of iron intake in order to provide dietary iron advice for individuals with low iron intake.

The key finding of the study conducted may be the observation that the assessed IRONIC-FFQ, despite the fact that it requires future assessments, conducted in other age groups of women, is characterised by a satisfactory level of validity and positively validated reproducibility in a group of young women. Moreover, it should be emphasised that the especially high level of reproducibility can be used in the dietary monitoring of patients in observations of the results of dietary interventions, including a change of iron intake. As a consequence, the IRONIC-FFQ may be indicated as a practical tool for the assessment of iron intake and for analysis of the results of dietary intervention in the anemia risk group of young women.

Despite the positively conducted validation, some limitations of the study must be indicated. On the one hand, there are the factors associated with the planned validation and its modifiable limitations, and these may be improved during further analysis of the IRONIC-FFQ. Yet, on the other hand, some of the limitations are general and for the time being would be difficult to overcome. The previously mentioned fact that the sample was not random but that individuals had volunteered for the study may be overcome by further validation in a randomly chosen group of women. Due to the fact that the dietary record may be imprecise, conducting a weighted record may improve its accuracy. Moreover, due to the general changes of one’s diet throughout the year, the repeated weighted records conducted during various seasons may be perceived as a valuable method for the assessment of the food frequency questionnaire regarding consumption during the previous year. Also, the fact that the dietary record and food frequency questionnaire were applied at a similar period of time may have influenced the results, so repeated dietary records and repeated IRONIC-FFQ should be applied during the analysed period but not directly one after another. However, an unmodifiable limitation is the fact that the dietary record is, in general, characterised by under-reporting associated with modifications of consumption during recording, so even a weighted record may not change it. Moreover, as food frequency questionnaires are generally prone to overestimating consumption, it is questionable how this inaccuracy could be removed, but it must be indicated, that dietary records are, in general, characterised by higher validity than food frequency questionnaires [47], so are a good reference method to use in validation.

On the other hand, the conducted validations had some important strengths that should also be indicated. These were associated with both the planned data collection and with the conducted statistical analysis. Conducting validation in a homogeneous group of young women who are healthy and have no modifications of diet, i.e., in a group that would especially benefit from routine iron intake assessment, is an applicable solution-focused approach. The obtained sample size was in accordance with recommendations on sample size for validation studies of food frequency questionnaires, as the guidelines state that at least 50 to 100 subjects for each demographic group is recommended [25]. The sample size was also approximate to the sample sizes chosen in other validation studies of iron food frequency questionnaires. In the study of Heath et al. [32], the study was conducted in a group of 49 female students; in the study of Beck et al. [10], it was conducted in a group of 115 women; in the study of Galante and Colli [37], in a group of 30 Internet users; in the study of Sichieri and Everhart [38], in a group of 88 university staff members; and in the study of Fayet et al. [36], in a group of 53 female adults.

The obtained questionnaire is a ready-to-use scheme that allows quick assessment of iron intake in young women. Moreover, the conducted validation, including various statistical methods, may be stated as being quite comprehensive, which allowed a comparison of the results with the results of various studies. The conducted discussion allows one to conclude that the obtained questionnaire may be a promising tool for iron intake assessment.

Conducting validation in comparison with the dietary record allows to obtain data of practical dietary intake, not disturbed by the applied supplementation, individual absorption, or loss (associated e.g., with menstrual bleeding). As the aim of the study was to obtain the valid and reproducible questionnaire to assess dietary iron intake, the validation in comparison with dietary records was essential. However, in the future, the further validation in comparison with iron status indicators may be planned, in order to assess the association between dietary iron intake assessed using the IRONIC-FFQ and iron status.

Taking into account the global target of the WHO for anemia prevention [5], obtaining a brief tool of iron intake assessment may be indicated as a priority. The validation presented above indicated that IRONIC-FFQ (presented in Table 1) is a tool characterised by a satisfactory level of validity and of positively validated reproducibility in a group of young women. As a consequence, the IRONIC-FFQ may be recommended for application as a practical brief method of assessment of iron intake adequacy in the population of young European women and may be a promising method for use in other populations.

## 5. Conclusions

(1) Assessment of the IRONIC-FFQ revealed a satisfactory level of validity and positively validated reproducibility.
(2) The IRONIC-FFQ may be indicated as a practical tool for the assessment of iron intake and for analysis of the results of dietary intervention in the anemia risk group of young women.

## Figures and Tables

**Table 1:** The IRONIC-FFQ (IRONIC-FFQ—IRON Intake Calculation-Food Frequency Questionnaire) form accompanied by the content of iron in one serving of a size specified in the IRONIC-FFQ questionnaire (information about the iron quantity in the serving was not placed in the IRONIC-FFQ).

Group of Products	Products	Serving Size	Iron Content/Serving (mg)
Meat	Liver (pork, beef, calf, poultry), pork kidney	100 g (palm of small hand)	13.30
	Other pork offal, poultry stomach	100 g (palm of small hand)	3.30
	Beef, calf, lamb, horse, goose, duck meat	100 g (palm of small hand)	2.60
	Pork meat	100 g (palm of small hand)	1.00
	Poultry meat	100 g (palm of small hand)	1.00
	Broth	250 g (1 glass)	0.25
Meat products	Blood pudding sausage	25 g (e.g., 1/2 of wiener, medium slice of ham, 5 slices of sausage)	4.22
	Other offal cold cuts	25 g (e.g., 1/2 of wiener, medium slice of ham, 5 slices of sausage)	1.35
	Loin cold cuts, ham, poultry sausages	25 g (e.g., 1/2 of wiener, medium slice of ham, 5 slices of sausage)	0.21
	Other sausages, wiener, smoked gammon, spam, pate, salami, brawn cold cut, bacon	25 g (e.g., 1/2 of wiener, medium slice of ham, 5 slices of sausage)	0.48
Eggs		50 g (1 egg)	1.10
Fish	Sardines	50 g (deck of cards)	1.07
	Other fish and fish products	50 g (deck of cards)	0.45
Dairy products	Milk and milk beverages (yoghurt, kefir, buttermilk, cream)	250 g (1 glass)	0.37
	Cottage cheese	50 g (1 thick slice, 2 tablespoons)	0.10
	Rennet and processed cheese	25 g (1 slice, 1 triangle serving)	0.15
Cereal products	White wheat and rye bread, bakery wares	35 g (1 slice, small roll)	0.37
	Dark bread, wholemeal, with grains, graham bread, pumpernickel bread	35 g (1 slice, small roll)	0.70
	Crispbread	10 g (1 slice)	0.40
	Wheat bran, wheat germs	10 g (1 spoon)	1.20
	Iron-fortified corn flakes and cereals	35 g (1 glass)	4.30
	Other cereal products (uncooked)	100 g (e.g., 1 glass of pasta or oatmeal, 1/2 glass of rice or groats)	2.70
Fruits	Fresh fruits	100 g (1 medium piece, 1 glass)	0.65
	Dried fruits	50 g (handful)	1.28
Vegetables	Dry legumes	100 g (1/2 of glass)	6.80
	Other vegetables	100 g (1 medium piece, 1 glass)	1.10
Potatoes		100 g (1 large piece)	0.50
Fats		10 g (1 spoon)	0.20
Nuts and seeds	Poppy, pumpkin and flaxseed	30 g (handful, 3 spoons of seeds)	3.78
	Other nuts and seeds	30 g (handful, 3 spoons of seeds)	1.28
Cocoa products	Cocoa	10 g (1 spoon)	1.07
	Chocolate	20 g (1/5 of bar)	0.41

**Table 2:** The iron intake calculated using a 3-day dietary record and the IRONIC-FFQ, accompanied by the share of individuals characterised by adequate or inadequate intake.

			3-Day Dietary Record	FFQ1	FFQ2
Mean (mg)			9.38	11.47	11.28
Standard deviation (mg)			3.54	5.18	5.14
Median (mg)			8.32 *	10.73 *	10.49 *
Minimum (mg)			3.31	3.01	2.44
Maximum (mg)			21.46	28.45	27.90
Share of individuals characterised in comparison with recommendation by Jarosz [30]	adequate intake	*n*	47	55	55
		(%)	62.7	73.3	73.3
	inadequate intake	*n*	28	20	20
		(%)	37.3	26.7	26.7

* distribution different than normal (verified using Shapiro–Wilk test—*p* ≤ 0.05); FFQ1—IRONIC-FFQ food frequency questionnaire filled out directly after conducting the three-day dietary record; FFQ2—IRONIC-FFQ food frequency questionnaire filled out 6 weeks after the FFQ1.

**Table 3:** The calculated contribution of product groups into the daily iron intake obtained from the IRONIC-FFQ conducted twice.

Group of Products	Mean ± Standard Deviation (%)	Median (%)	Minimum–Maximum (%)
Meat	10.65 ± 9.17	8.29 *	0–58.50
Meat products	6.23 ± 6.72	4.13 *	0–46.92
Eggs	4.86 ± 3.37	4.88 *	0–24.72
Fish	1.23 ± 1.52	0.79 *	0–8.05
Dairy products	3.90 ± 2.21	3.51 *	0–14.10
Cereal products	32.31 ± 12.78	30.35 *	5.43–73.37
Fruits	8.12 ± 5.59	7.29 *	0–36.88
Vegetables including dry legumes	19.30 ± 11.69	18.37 *	0–55.89
Potatoes	2.56 ± 2.60	1.92 *	0–16.76
Fats	0.16 ± 0.29	0.11 *	0–3.29
Nuts and seeds	7.90 ± 7.73	6.17 *	0–35.94
Cocoa products	2.77 ± 3.00	1.97 *	0–19.32

* Distribution different than normal (verified using Shapiro–Wilk test—*p* ≤ 0.05).

**Table 4:** The number and share of individuals misclassified and classified into the same tertile, as well as individuals of the same or conflicting iron intake adequacy category.

			FFQ1 vs. 3-Day Dietary Record	FFQ1 vs. FFQ2
Individuals classified into the same tertile		*n*	40	59
		%	53.3	78.7
Individuals classified into adjacent tertiles		*n*	26	14
		%	34.7	18.7
Individuals misclassified (classified into opposite tertiles)		*n*	9	2
		%	12.0	2.7
Weighted κ statistic			0.36	0.73
Individuals of the	same iron intake adequacy category	*n*	49	67
		%	65.3	89.3
	conflicting iron intake adequacy category	*n*	26	8
		%	34.7	10.7

FFQ1—IRONIC-FFQ food frequency questionnaire filled out directly after conducting the three-day dietary record; FFQ2—IRONIC-FFQ food frequency questionnaire filled out 6 weeks after the FFQ1.

## Abbreviations

FFQ	Food Frequency Questionnaire
IRONIC-FFQ	IRON Intake Calculation-Food Frequency Questionnaire
WHO	World Health Organization
LOA	Limits of Agreement Value
